# Cigarette smoke, but not novel tobacco vapor products, causes epigenetic disruption and cell apoptosis

**DOI:** 10.1016/j.bbrep.2020.100865

**Published:** 2020-11-21

**Authors:** Naoko Hattori, Takeya Nakagawa, Mitsuhiro Yoneda, Kaori Nakagawa, Hiromi Hayashida, Takashi Ito

**Affiliations:** a Nagasaki University Graduate School of Biomedical Sciences, Nagasaki, Japan; b Department of Biochemistry, Nagasaki University School of Medicine, Nagasaki, Japan

**Keywords:** Epigenetics, EGR1, Transcription

## Abstract

Heat-Not-Burn (HNB) products, generating vapor without combusting tobacco leaves, have been developed with the expectation that the number and quantity of chemicals in the vapor of these products would be reduced compared with the smoke from conventional combustible cigarettes. However, whether the lower chemical levels correlate with lower toxicity remains to be determined.

Here we examined differences in the biological effects of conventional cigarette smoke (CS) and two HNB products, Ploom TECH and Ploom TECH+, using the cultured cancer cell line A549 and the normal bronchial epithelium cell line BEAS-2B. The conventional CS 3R4F extract (0.5%) markedly decreased cell proliferation of both A549 and BEAS-2B cells; however, 0.5% extracts of these commercially available HNB products did not affect cell growth. To determine the cause of decreased cell proliferation, a TUNEL assay was performed, and the results indicated that apoptosis had occurred in both A549 and BEAS-2B cells at 24 h after exposure to 3R4F. To further explore the effect of CS on epigenetics, we performed western blotting to detect histone H2A phosphorylation, which is known to affect transcriptional regulation. Only the 3R4F extract decreased histone H2A phosphorylation in both A549 and BEAS-2B cells. Next, we examined alterations in gene expression after treatment of A549 cells with Ploom TECH, Ploom TECH+, or 3R4F extracts. It was found that 339, 107, and 103 genes were upregulated more than 2 fold in A549 cells treated with 3R4F, Ploom TECH, or Ploom TECH + extracts, respectively. Among the 339 genes that were upregulated in response to 3R4F, we focused on *EGR1*, *FOS*, and *FOSB*, since they were upregulated more than 100 fold, which was confirmed using RT-qPCR. These results suggest that CS, but not HNB products, cause epigenetic disruption and cell apoptosis, possibly by elevating transcription of genes such as *EGR1*.

## Highlights

• 3R4F cigarette smoke (CS) but not Ploom TECH or Ploom TECH+ Heat-Not-Burn (HNB) repressed cell proliferation.
• 3R4F but not the Ploom TECH or Ploom TECH+ induced cell apoptosis.
• 3R4F but not the Ploom TECH or Ploom TECH+ caused abnormal histone modifications.
• 3R4F caused more gene alterations than the Ploom TECH or Ploom TECH+.
• 3R4F but not the Ploom TECH or Ploom TECH+ caused upregulation of the *EGR1*, *FOS*, and *FOSB* genes.

## 1 Introduction

The use of tobacco-containing vapor products is increasing worldwide. In addition to e-cigarettes, which produce vapor by heating a nicotine-containing liquid, Heat-Not-Burn (HNB) products, in which the tobacco is heated without being combusted, have been developed [1]. Emissions from HNB products have been reported to contain lower levels of harmful chemicals than conventional cigarette smoke (CS), [2]. However, whether this reduction of potentially harmful chemicals is correlated with decreased toxicity and lower risk for developing chronic obstructive pulmonary disease (COPD) or other disorders needs to be verified.

COPD is a chronic, irreversible respiratory disease that is caused by an abnormal inflammatory response of the lungs to noxious particles or gases, resulting in chronic bronchiolitis and emphysema, which cause airflow limitation [3]. Although miscellaneous environmental and genetic risk factors are involved in the pathogenesis of COPD, CS is regarded as one of the most important [4]. The primary target of inhaled CS is the airway epithelium, which is a barrier to inhaled toxic chemicals. Several mechanisms are possible for COPD development, such as an influx of inflammatory cells into the airway, resulting in chronic inflammation, or an imbalance between proteolysis and anti-proteolysis, resulting in lung tissue destruction and oxidative stress. In addition, apoptosis of structural cells in the lung is regarded as an important event in the pathogenesis of COPD. An increase in apoptotic alveolar epithelial and endothelial cells is observed in the lungs of COPD patients, suggesting a role for this process in the destruction of lung tissue and the development of emphysema and COPD [5].

To understand the mechanisms for how CS contributes to COPD development, several approaches have been developed. Capturing the smoke emitted from a cigarette by bubbling it through an aqueous solution is one approach. Such preparations (known as smoking extracts) have been widely used as sample sources for various purposes, such as in vitro assays. That smoking extracts differ from the untreated smoke is a limitation. However, in vivo, bronchial epithelial cells are not directly exposed to the smoke itself but rather to components that have been extracted into biologically secreted fluids. Therefore, in vitro cell culture studies using smoke preparations remain important tools in the assessment of CS-induced toxicity [6].

In this study we examined differences in the biological effects of conventional CS and two commercial HNB products, Ploom TECH and Ploom TECH+, using the lung cancer cell line A549 and the normal bronchial epithelium cell line BEAS-2B. Conventional 3R4F extracts (0.5%) from CS markedly decreased cell proliferation of both A549 and BEAS-2B cells, while 0.5% extracts of Ploom TECH or Ploom TECH + did not affect cell growth. TUNEL assays indicated that apoptosis was present in both A549 and BEAS-2B cells at 24 h after exposure to 3R4F but not after exposure to Ploom TECH or Ploom TECH+. Alterations of gene expression in A549 cells in which *EGR1*, *FOS*, and *FOSB* genes were upregulated by more than 100 fold occurred only after 3R4F treatment. These results suggest that CS, but not HNB products, causes epigenetic disruption and cell apoptosis, possibly by elevating expression of genes such as *EGR1*.

## 2 Material and methods

### 2.1 Smoke and aerosol generation

Smoking extracts were prepared as previously described [1]. Briefly, 3R4F cigarettes were purchased from the University of Kentucky, Kentucky Tobacco Research and Development Center (Lexington, KY, USA). Two commercial HNB products, Ploom TECH and Ploom TECH+, were obtained from Japan Tobacco Inc. (Tokyo, Japan). HNB products consist of three modules: a puff-activated electrical heating device with a rechargeable battery, a cartomizer containing a nicotine-free carrier liquid, and a tobacco capsule containing granulated tobacco. Untreated 3R4F cigarette smoke and aerosols from Ploom TECH or Ploom TECH + HNB products were generated by machine smoking using Health Canada Intense (HCI) puffing conditions (55 mL puff volume, 2-s duration, 30-s puff interval, and bell-shaped puff profile). The 3R4F cigarette was smoked until the remaining butt length was 35 mm. For the HNB products, the total puff number for each aerosol collection per tobacco capsule was set at 70, which was based on the product specification. Aerosols were resolved in DMSO at a concentration of 40 mg/ml for 3R4F extract and 100 mg/ml for both Ploom TECH and Ploom TECH + extracts.

### 2.2 Cell culture and growth curve

A549 and BEAS-2B cells were cultured in a humidified chamber (37 °C, 5% CO_2_ in air) with Roswell Park Memorial Institute (RPMI), supplemented with 10 μg/ml gentamicin and 10% fetal calf serum (Life Technologies). One day before counting, the cells were seeded into 24-well plates at a density of 0.4 × 10^4^ cells/cm^2^. On the next day, the cells were treated with 0.5% DMSO, Ploom TECH extract, Ploom TECH + extract, or 3R4F extract in duplicate, and the day was designated as day 0. From each well, four images were collected on days 0, 1, 2, and 3, and the cells were counted within a 1-mm^2^ square for each image.

### 2.3 Western blotting

Lysates were prepared using a sodium dodecyl sulfate (SDS) sample buffer, separated by SDS-PAGE (7% or 12.5%), transferred to a nitrocellulose membrane (Bio-Rad), and blocked with 3% BSA in TBST (Tris-buffered saline containing 0.05% Tween-20). To detect cyclin D1 and H2ApT120, membranes were incubated with anti-cyclin D1 (Abcam Ab134175) or anti-H2ApT120 [7] antibodies, followed by horseradish peroxidase-conjugated anti-rabbit antibodies (Sigma cat. no. A0545), and the subsequent chemiluminescence was obtained using SuperSignal West Femto Maximum Sensitivity Substrate (Thermo Fisher Scientific, Waltham, MA). To detect hTBP, membranes were incubated with anti-TBP antibodies [8], followed by washing and subsequent incubation with protein A conjugated with Alexa Fluor™ 647 (Thermo Fisher Scientific, Waltham, MA, cat. no. p21462). Fluorescence signals were captured by employing a Typhoon™ FLA 9000 imager (GE Healthcare) to scan the probed membranes, with the PMT setting set at 1000 V.

### 2.4 TUNEL assay and FACS analysis

A549 and BEAS-2B cells were treated with 1 μM stauropsprine and 0.5% DMSO, Ploom TECH extract, Ploom TECH + extract, or 3R4F extract, as indicated. Twenty-four hours after treatment, the cells were subjected to a TUNEL assay according to the manufacturer's instructions (DeadEnd Fluorometric TUNEL System, Promega). A549 and BEAS-2B cells were stained with propidium iodide and analyzed using a FACSCanto II flow cytometer.

### 2.5 RNA-seq

RNA-seq was performed as previously described [9]. Total RNA was extracted using ISOGEN II reagent (Nippon Gene), according to the manufacturer's protocol. RNA quality was confirmed using the Agilent RNA 6000 Nano kit with an Agilent 2100 Bioanalyzer instrument (Agilent Technologies). RNA-seq libraries were generated using the TruSeq Stranded mRNA LT Sample Prep kit (Illumina) and sequenced with the MiSeq system (Illumina). Sequences were mapped to the human genome (GRCh38) with the Illumina Analysis Pipeline. The gene expression level was quantified by the number of uniquely mapped reads per kilobase of exon per million mapped reads (RPKM) using the Partek® Genomics Suite. Gene expression level determination and gene ontology (GO) analysis were also performed with the Partek® Genomics Suite. Significantly enriched GO functional groups were defined as having an enrichment score ≥3 (P value < 0.05). All RNA-seq data can be found online in the NCBI GEO submission (GSE157165).

### 2.6 RTqPCR

cDNA was created from 0.5 μg total RNA using an oligo(dT)primer (Life Technologies), random hexamers (Takara), and M-MuLV reverse transcriptase (NEB). Using SYBR green and appropriate master-mix reagents, real-time RT-PCR was performed with an ABI PRISM 7900HT instrument (Applied Biosystems). Target gene expression levels were normalized via *GAPDH* expression, and the relative expression level was calculated by comparing with the DMSO control. The PCR primers used were:

*EGR1*
  (forward) 5′-AGGCGGCGATTTTTGTATGT-3′,
  (reverse) 5′-GGGCAATAAAGCGCATTCAA-3′;
*FOS*
  (forward) 5′-TTATTTATTAAGATGGATTCTCAG-3′,
  (reverse) 5′-CTTGGAACAATAAGCAAACAATGC-3′;
*FOSB*
  (forward) 5′- AGTGAGACTGAGGGATCGTAGA-3′,
  (reverse) 5′- GGGGTCGGGGATTCATTGAA-3′;
*GAPDH*
  (forward) 5′-GGAGCGAGATCCCTCCAAAAT-3′,
  (reverse) 5′- GGCTGTTGTCATACTTCTCATG -3′.

## 3 Results

### 3.1 *3R4F but not Ploom TECH or Ploom TECH + extracts repress cell proliferation of both A549 and BEAS-2B cells*

**Fig. 1:**
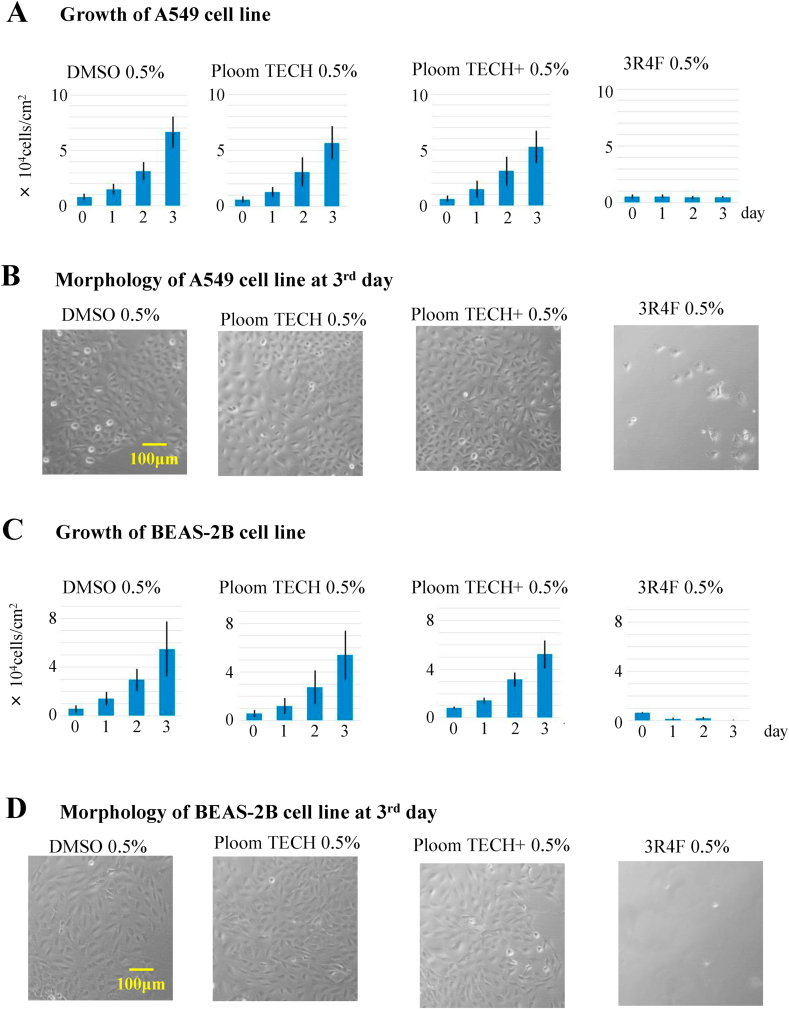
3R4F extract but not Ploom TECH or Ploom TECH + extracts repressed cell proliferation in both A549 and BEAS-2B cells. A549 cells and BEAS-2B cells were plated at a concentration of 0.4 × 10^4^ cells/cm^2^ in 24-well plates. Twenty-four hours after plating, DMSO, Ploom TECH extract, Ploom TECH + extract, or 3R4F extract were added at a concentration of 0.5% on day 0 in duplicate. For each well, four images from one well were collected on days 0, 1, 2, and 3. The cells were counted within a 1-mm^2^ square for each image, and cell growth was evaluated for A549 cells (A) and BEAS-2B cells (C). Representative morphology on day 3 for A549 cells (B) and BEAS-2B cells (D) treated with the indicated extracts. Duplicated experiments were done three times, and the results were found to be consistent.

In order to examine the extent to which smoking extracts cause cell damage, the cultured cancer cell line A549 and the normal bronchial epithelium cell line BEAS-2B were used. Both cell lines were plated at a concentration of 0.4 × 10^4^ cells/cm^2^ using 24-well plates. Twenty-four hours after plating, DMSO, Ploom TECH extract, Ploom TECH + extract, or 3R4F extract were added at a final concentration of 0.5% on day 0. The 3R4F extract markedly repressed cell proliferation of both A549 and BEAS-2B cells while killing most of the cells, but neither Ploom TECH nor Ploom TECH + affected cell growth (Fig. 1A, C). From the morphology on day 3, almost all cells treated with 3R4F extract had died (Fig. 1B, D). It was previously reported that a lower concentration of CS extract induced apoptosis, and a higher concentration of CS extract induced necrosis [10]. Therefore, we examined whether CS extract induced apoptosis or necrosis in our assay.

### 3.2 *3R4F but not Ploom TECH or Ploom TECH + extracts induced apoptosis in both A549 and BEAS-2B cells*

**Fig. 2:**
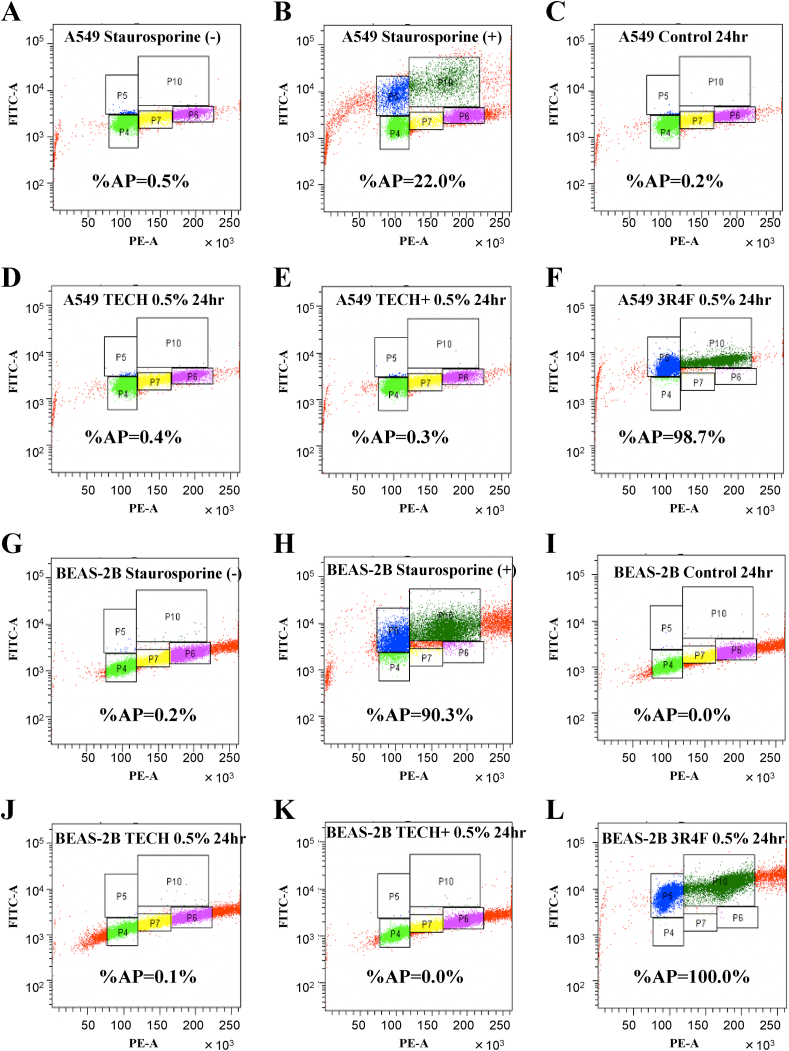
3R4F extract but not the Ploom TECH or Ploom TECH + extracts induced apoptosis in both A549 and BEAS-2B cells. A549 (A–F) and BEAS-2B (G–L) cells were untreated (A, G) or treated (B, H) with 1 μM staurosporine, 0.5% DMSO (C, I), 0.5% Ploom TECH extract (D, J), 0.5% Ploom TECH + extract (E, K), or 0.5% 3R4F extract (F, L) and analyzed using a flow cytometer after treatment using the DeadEnd™ fluorometric TUNEL system according to the manufacturer's protocol. %AP, the percentage apoptosis, calculated as (P5+P10)/(P4+P5+P6+P7+P10) x 100.

To detect the presence of apoptosis, a TUNEL assay was performed (Fig. 2). For a positive control, staurosporine, which is known to induce apoptosis, was used. Twenty-four hours after adding 1 μM staurosporine with 0.5% DMSO, Ploom TECH extract, Ploom TECH + extract, or 3R4F extract, the cells were collected, and DNA fragmentation was examined using the DeadEnd Fluorometric TUNEL System, Promega. Apoptosis had been induced in ~22% of the A549 lung cancer cells and 90.3% of BEAS-2B cells at 24 h after staurosporine treatment when compared with no treatment (Fig. 2A, B, G, H). Consistent with previously observation using a lower concentration of CS extract [10], apoptosis was induced in ~98.7% of the A549 lung cancer cells and 100.0% of the BEAS-2B cells at 24 h after treatment with 0.5% 3R4F (CS) extract (Fig. 2F, L). However, apoptosis was induced in <0.5% of the of the A549 lung cancer cells and <0.5% of BEAS-2B cells at 24 h after treatment with 0.5% DMSO, Ploom TECH extract, or Ploom TECH + extract (Fig. 2C–E, I-K). These findings corroborate the previous report that chemical analysis of the HNB aerosol demonstrated that levels of Hoffmann analytes, including acetaldehyde, acrolein, carbon monoxide, benzene, 1,3-butadiene, formaldehyde, N-nitrosonornicotine (NNN), N-nitrosonornicotine ketone (NNK), and benzo[a]pyrene (B[a]P), were substantially lower or below quantifiable levels compared with 3R4F (CS) extract. Results from in vitro cell culture experiments clearly indicated that, in contrast to 3R4F extract, the Ploom TECH and Ploom TECH + extracts failed to induce apoptosis.

3R4F but not Ploom TECH or Ploom TECH + extracts caused abnormal histone modification, which is known to be correlated with changes in gene transcription.

**Fig. 3:**
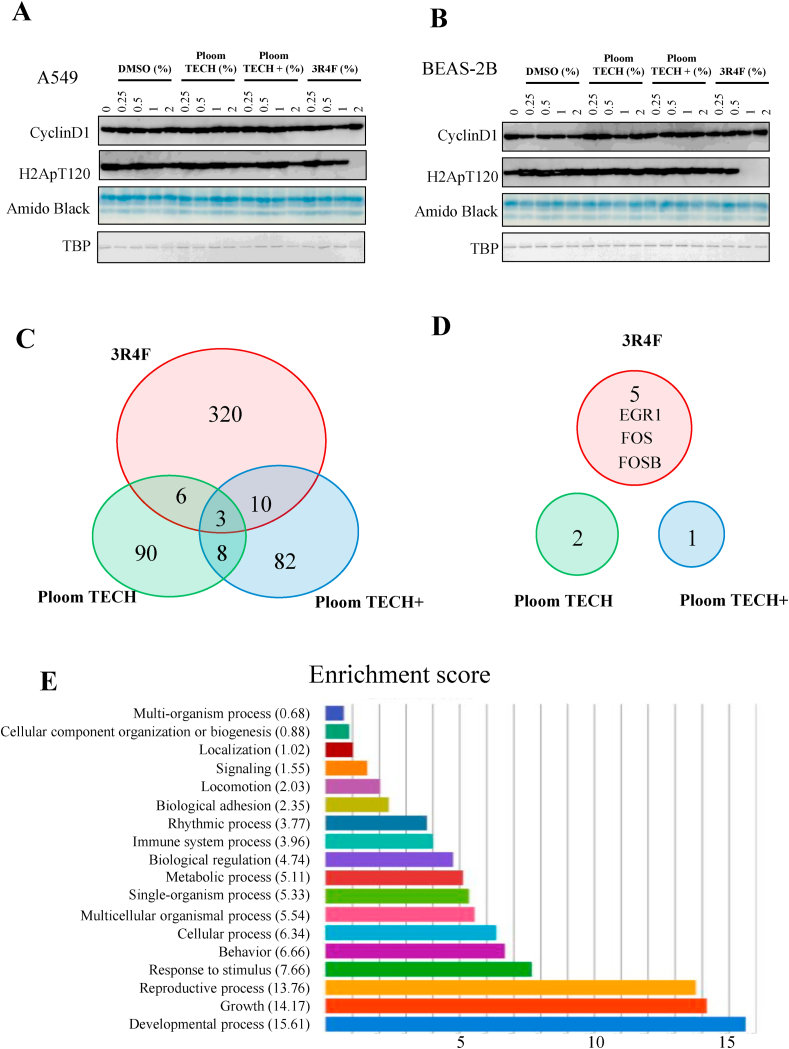
3R4F extract caused abnormal histone modifications and more gene alterations than the Ploom TECH or Ploom TECH + extracts. To HeLa cells (A) and BEAS-2B (B) cells were added 0.25, 0.5, 1, and 2% DMSO, Ploom TECH extract, Ploom TECH + extract, or 3R4F extract. Twenty-four hours after treatment, the cells were collected and subjected to western blotting for cyclin D1, H2AT120 phosphorylation (H2ApT120), and TBP. Amido black staining and anti-TBP western blotting indicated the amount of sample loaded. Venn diagram representing the overlap among 2-fold-upregulated genes (C) and 100-fold-upregulated genes (D) induced by Ploom TECH extract, Ploom TECH + extract, and 3R4F extract treatment of A549 cells. The numbers of the genes are represented. (E) Gene ontology analysis of genes upregulated by treatment with 3R4F extract.

To determine the underlying mechanisms of 3R4F extract-induced apoptosis, we investigated histone modification, which is an important variable in epigenetics. The histone H2A C-terminus is flexible and protrudes from the nucleosome core, as do the histone N-terminal domains. It is known that the C-terminal end of histone H2A and the N-terminal end of histone H3 contain the linker DNA entry and exit points, suggesting their functional importance [11]. We previously found that hVRK1 phosphorylates nucleosomal H2A (T120) around the promoter region of genes such as *CCND1* and regulates transcription [7,12]. Therefore, we investigated histone H2A (T120) phosphorylation and cyclin D1 expression using western blotting. Four hours after treatment with 0.25, 0.5, 1, or 2% DMSO, Ploom TECH extract, Ploom TECH + extract, or 3R4F extract, the cells were collected, and western blotting was performed. A two-percent 3R4F extract treatment of A549 cells and a 1 or 2% 3R4F extract treatment of BEAS-2B cells, but not treatment with Ploom TECH or Ploom TECH + extracts, caused a loss of bulk histone H2A (T120) phosphorylation (Fig. 3A and B). However, this loss of phosphorylation reflects bulk core histone H2A but not H2A around the promoter region. Since cyclin D1 is regulated by histone H2A phosphorylation of the promoter region, it is possible that loss of bulk histone H2A phosphorylation does not reflect the phosphorylation of a specific promoter [7]. Although loss of bulk core histone H2A phosphorylation is strongly correlated with 3R4F extract treatment, the mechanisms of apoptosis induction remain to be determined.

Treatment with 3R4F extract caused more gene alterations than Ploom TECH or Ploom TECH + extracts.

**Fig. 4:**
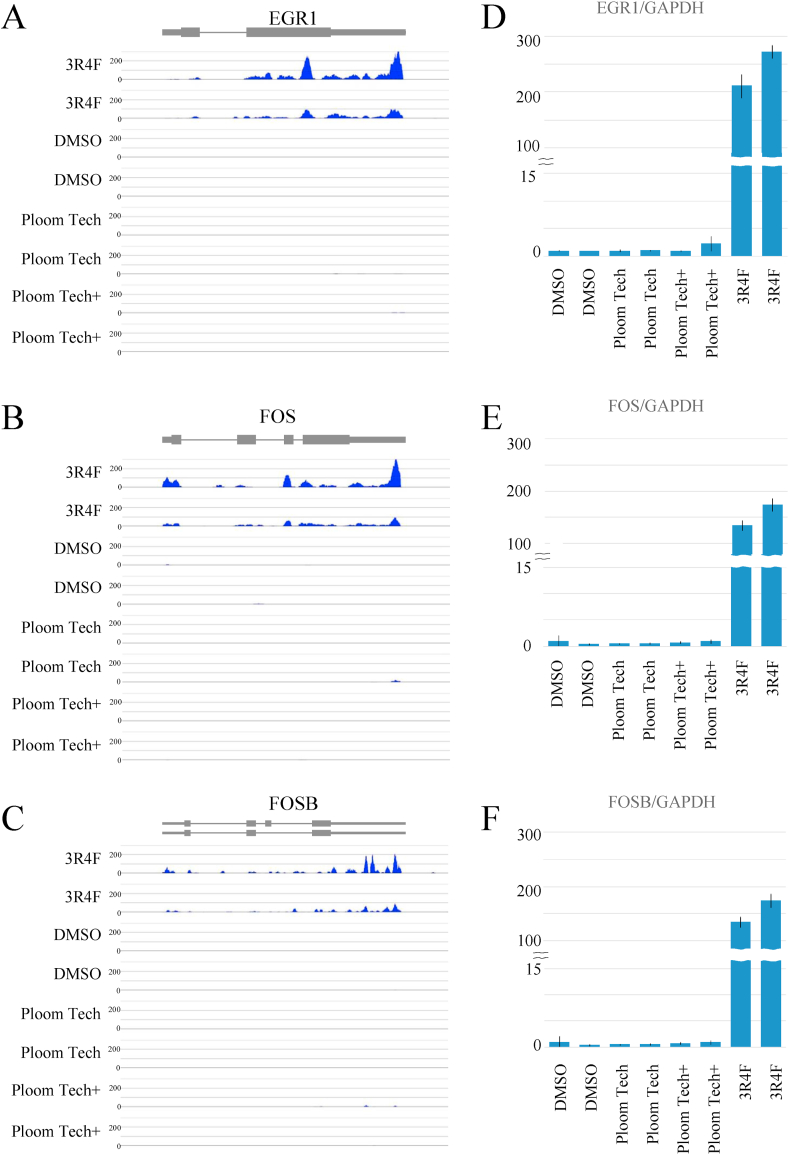
3R4F extract but not the Ploom TECH or Ploom TECH + extracts caused upregulation of the EGR1, FOS, and FOSB genes. (A, B, C) RNA-seq signal tracks around the *EGR1*, *FOS*, and *FOSB* genes in A549 cells, which were collected 4 h after treatment with 2% DMSO, 2% Ploom TECH extract, 2% Ploom TECH + extract, or 2% 3R4F extract. (D, E, F) RT-qPCR results for the *EGR1*, *FOS*, and *FOSB* genes using the same RNA. The relative expression was determined after adjusting for *GAPDH* expression and comparing with the DMSO control.

To investigate the gene alterations that might be correlated with cell apoptosis, an RNA-seq experiment was performed after treatment of A549 cells with Ploom TECH, Ploom TECH+, or 3R4F extracts. Venn diagrams of genes upregulated 2 fold (Fig. 3C) or 100 fold (Fig. 3D) are shown. The 3R4F extract caused more gene alterations than Ploom TECH or Ploom TECH + extracts. GO analysis of the genes affected by 3R4F identified genes involved in developmental processes as well as growth and reproductive processes (Fig. 3E). Among the genes upregulated 100 fold, only *EGR1*, *FOS*, and *FOSB* showed a significant RNA-seq profile (Fig. 4A–C). Expression of these three genes was confirmed using RT-qPCR (Fig. 4D–F). 3R4F extract but not Ploom TECH or Ploom TECH + extracts caused significant activation of the *EGR1*, *FOS*, and *FOSB* genes. Previously, it was found that *EGR1* is activated by CS extract; however in this experiment we found that *EGR1*, *FOS*, and *FOSB* are all activated by 3R4F but not by Ploom TECH or Ploom TECH+.

## 4 Discussion

In this study it was found that conventional cigarette smoke (CS) but not the Ploom TECH or Ploom TECH + HNB products cause apoptosis, a change in histone H2A phosphorylation that is closely correlated with changes in gene expression, and upregulation of the *EGR1*, *FOS*, and *FOSB* genes. Among these upregulated genes, we focused on *EGR1,* since it has multiple functions relating to the development of cancer, cardiovascular disease, memory and psychiatric disorders, and apoptosis [[13], [14], [15], [16], [17]]. For the development of cancer, observation over long periods is required, and for cardiovascular disease or memory and psychiatric disorders, functional changes need to be assessed in complex organs. By contrast, in cultured cell experiments, apoptotic mechanisms, which may involve EGR1, can be readily investigated.

Previously, it was reported that, in human lung fibroblasts, CS extract stimulates EGR1 expression and subsequent metalloproteinase 2 (MMP-2) activation. These results support the hypotheses that perturbing the balance of protease–antiprotease activity may result in the destruction of the alveolar septal architecture and the development of CS-related COPD [18]. On the other hand, it has also been reported that EGR1 induces apoptosis in multiple situations [[19], [20], [21], [22]]. Among several mechanisms of COPD development, the apoptosis of structural cells in the lung is regarded as an important event in its pathogenesis [5,23,24]. In either case, EGR1 upregulation by CS may play a role in the development of COPD.

This report, that cigarette smoke (CS) but not Ploom TECH or Ploom TECH + HNB products causes apoptosis and a change in transcription-related histone modifications and is highly correlated with upregulation of *EGR1*, suggests that CS, but not HNB products, causes COPD via apoptotic mechanisms. However, this suggestion is based on in vitro experiments; to make a stronger conclusion, in vivo animal experiments or human clinical trials would be required.

## Declaration of competing interest

The authors have no conflicts of interest to declare.
